# Primary endoscopic nasopharyngectomy for nasopharyngeal carcinoma: systematic review & meta-analysis

**DOI:** 10.3389/fsurg.2026.1779795

**Published:** 2026-06-26

**Authors:** Siyuan Ding, Leo Li, Jenny Lee, Brian Mak, Christopher Liao, Andy Chan, Levina Li, Calvin Lai, Samuel Chow, Jason Chan, David Yeung

**Affiliations:** 1 Department of ENT, Cambridge University Hospitals NHS Foundation Trust, Cambridgeshire, United Kingdom; 2 Department of Otorhinolaryngology, Head and Neck Surgery, The Chinese University of Hong Kong, Sha Tin, Hong Kong SAR, China

**Keywords:** endoscopic, head and neck, nasopharyngeal carcinoma, nasopharyngectomy, surgery, undifferentiated

## Abstract

**Objective:**

A systematic review and meta-analysis for the use of primary endoscopic nasopharyngectomy (pENPG) alone in the treatment of primary nasopharyngeal carcinoma (pNPC). To evaluate its benefits and limitations compared to current standard therapy.

**Methods:**

A literature search was conducted in January 2025 using PubMed, Embase, MEDLINE, and Cochrane databases. Studies were selected based on strict inclusion/exclusion criteria. Data was extracted on selection criteria for surgery, operation details, post-operative complications and treatment outcomes (survival outcomes and quality of life). Data was analysed using qualitative and quantitative methods and meta-analysis was performed as appropriate.

**Results:**

Four studies involving 164 patients who underwent pENPG were identified. All studies were retrospective in nature. Two studies analysed pENPG alone, whereas the other two studies compared pENPG to intensity-modulated radiotherapy (IMRT) or open surgery. 58% of patients who underwent pENPG had localised stage I NPC (T1N0M0). Three and five-year overall survival (OS) for pENPG-treated stage I NPC was 100% in two studies. Meta-analysis for all tumour stages yielded a pooled 5-year OS of 90.6% for pENPG (± chemoradiotherapy). One study compared pENPG vs. IMRT for stage I disease, with 5-year OS at 100% and 99.1% respectively. Radiotherapy-associated toxicities were significantly reduced with pENPG only. The severity of surgical complications correlated with the extent of resection.

**Conclusions:**

pENPG utilisation is rare in current practice. It may be a feasible alternative to IMRT for early stage disease to minimise radiotherapy toxicities but prospective studies are necessary to evaluate this further.

## Introduction

Nasopharyngeal carcinoma (NPC) is known to have a predilection to areas of Southern China and Southeast Asia (1), particularly the undifferentiated type (2). This non-keratinising type of NPC is closely associated with Epstein–Barr virus (EBV) infection (2). NPC is generally diagnosed at the late stage due to difficulties in detection, aggressive nature of disease and high rates of nodal metastasis (3, 4). Therefore, only a minority of patients present with early-stage or localised pNPC (stage I and II) (5). However, improvements in the identification of symptoms, early detection using EBV-DNA (for the undifferentiated type) and advances in imaging have reduced the rates of advanced stage disease (6).

The mainstay of treatment for stage I pNPC is IMRT, which achieves excellent rates of control and five-year disease-free survival (DFS) of over 90% (7, 8). Locally advanced (stage III to IVa) pNPC is usually treated with concurrent chemotherapy (7), providing 5-year OS rates of around 80% (9). However, IMRT can be associated with significant radiotherapy toxicities. Commonly described late toxicities after IMRT for NPC range from low-grade neck fibrosis and xerostomia to clinically significant dysphagia (requiring tube feeding), hearing loss, temporal lobe necrosis and osteoradionecrosis, often associated with higher radiation doses and late stage disease (10). Normal tissue complication probability (NTCP) models suggest a combination of radiation, disease and patient factors contribute to toxicity (11, 12). Lower doses of radiotherapy can mitigate against these toxicities, but at the cost of a higher rate of recurrence (13, 14).

Primary surgery would avoid radiotherapy-associated toxicities, so it is important to evaluate whether this approach would be a viable alternative to IMRT. Currently, surgery is mostly limited to taking tissue samples or as salvage treatment for patients with recurrent NPC (rNPC) (15–18). The use of pENPG is far more limited in the current literature. However, with advances in endoscopic surgery and expertise, pENPG is becoming increasingly realistic (19). To the best of our knowledge, no systematic review has been conducted which synthesizes the existing evidence on the use of pENPG. This study aims to assess the quality of the literature, examine the selection criteria and operative approaches used and evaluate the clinical outcomes with pENPG.

## Methods

### Study selection

The systematic review was conducted in accordance with the Preferred Reporting Items for Systematic Reviews and Meta-Analyses (PRISMA) statement (20).

The systematic review was designed according to the Participant, Intervention, Comparison, Outcome, and Study design (PICOS) framework.

1. Population: Humans diagnosed with pNPC
2. Intervention: Treatment with pENPG
3. Comparison: Treatment with radiotherapy or non-pENPG methods
4. Outcome: Survival data, safety, efficacy, adverse effects
5. Study design: Publication types included in the search criteria included randomized control trials, case-control studies, cohort studies, case series and case reports, but not abstracts, conference articles or meeting communications.

Studies were selected based on the following inclusion criteria:

1. Patients must be diagnosed with pNPC as defined by the WHO classification (21).
2. Some or all study participants were treated with solely pENPG.
3. Includes information on follow-up and survival outcomes.
4. Primary research including randomized control trials, case-control studies, cohort studies, case series and case reports.
5. Articles available in English.

Studies that were excluded include:

- Non-original articles such as systematic and literature reviews.
- Preprints, conference abstracts or other studies with insufficient data.

### Search strategy

An electronic search was conducted on the PubMed, Embase, MEDLINE and Cochrane library databases in January 2025. The search strategy was as follows:

- (Endoscopy OR endoscopic).ti,ab.
- (nasopharyngectomy OR nasopharynx OR nasopharyngeal OR transnasal).ti,ab.
- (primary).ti,ab.
- 1 & 2 & 3

**Figure 1:**
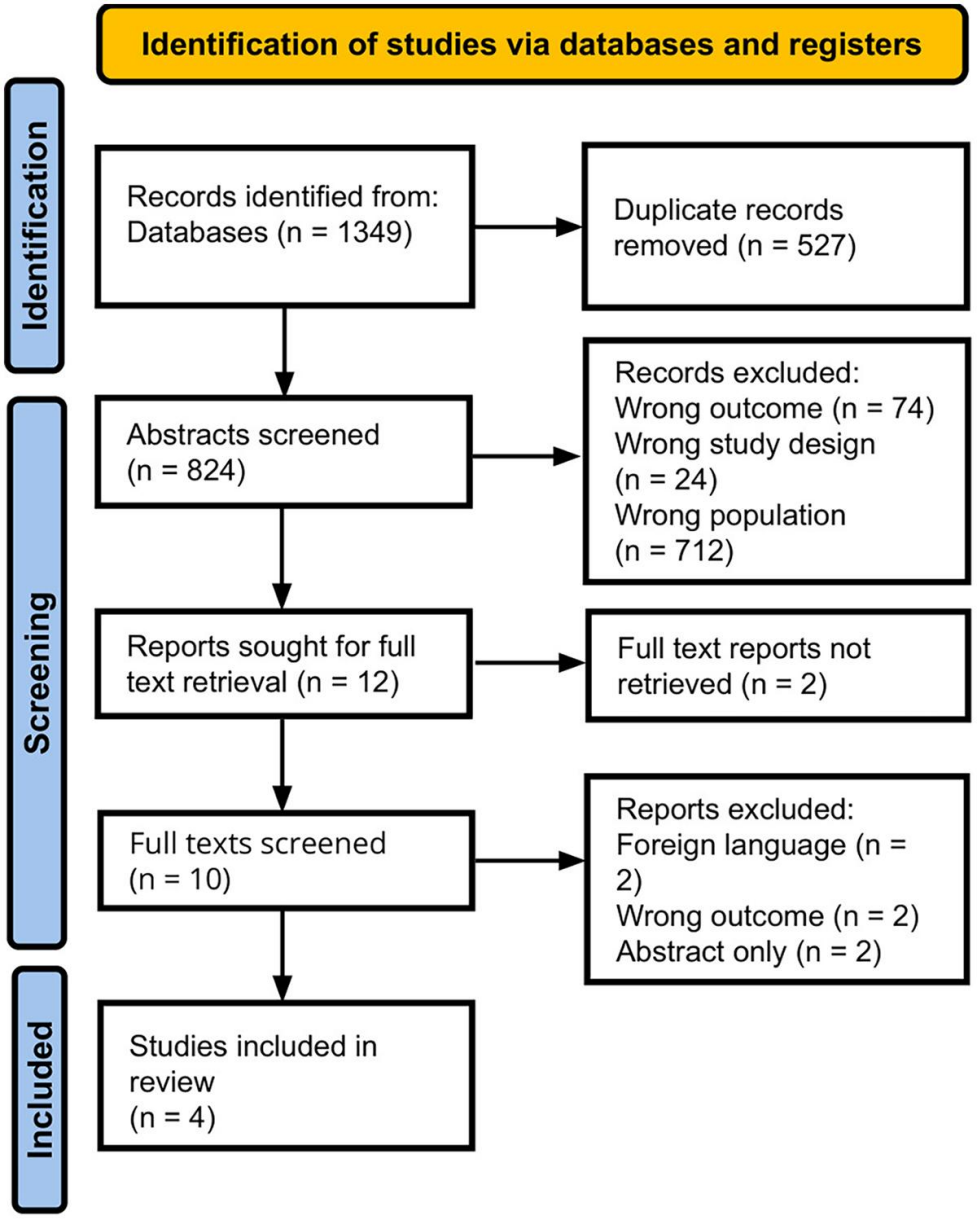
PRISMA flowchart.

In total, 1,349 articles were identified across all databases. After de-duplication, 824 articles remained. Abstracts were independently screened by two reviewers (SD and DCMY) and excluded if they included the wrong outcome, study design or target population. Discrepancies between the reviewers were assessed and resolved. 12 studies were selected for full-text review. Four studies ultimately met the inclusion criteria and were included in the review. The screening process is summarised in a PRISMA flowchart (Figure 1).

### Risk of bias assessment

The quality of included studies were assessed using two different quality assessment tools. The Risk of Bias in Nonrandomized Studies of Interventions (ROBINS-I) tool was used for non-randomised interventional studies with at least two different comparison arms. The NIH Quality Assessment Tool for Case Series Studies was used for case reports and case series. Quality of studies were independently assessed by two reviewers (SD and DCMY) and disparities were resolved.

### Data extraction

Data was tabulated using Microsoft Excel. Background information was collected on study design and treatment arms. Patient age and sex were recorded if available. Associated symptoms, size and dimensions of tumour, anatomical subsite, tumour stage and histological data were documented if available. Operative details were noted including selection criteria for surgery, time to surgery from diagnosis, operation length, intraoperative bleeding volume and operative method. Primary outcomes included three or five-year overall survival (OS) and disease-free survival (DFS). Secondary outcomes included post-operative complications and other measures of survival, such as local relapse-free survival (LRFS), regional relapse-free survival (RRFS), distant metastasis-free survival (DMFS) and disease-specific survival (DSS).

### Statistical analysis

Data was assessed for suitability in meta-analyses based on similarity of study design, study participants, methodology and measured outcomes. A meta-analysis of proportions was performed to analyse five-year OS following pENPG using a Freeman-Tukey double arcsine transformation to stabilise variances. To compare five-year OS between pENPG and IMRT groups, a meta-analysis of dichotomous variables was conducted. Risk ratios (RRs) were used to compare the efficacy between the two interventions. Study weights for each meta-analysis was calculated using the inverse-variance method. Proportions, 95% confidence intervals (CIs) and study weights were visualised using forest plots. Heterogeneity was assessed using the Tau^2^ statistic, Cochran's *Q* test and I^2^ statistic. Funnel plots and Egger's regression were not used due to the small number of included studies. Results were pooled using a random effects model and pooled estimates and confidence intervals were presented as a diamond in the forest plots. The meta-analysis of proportions was conducted in R (version 4.3.1) using the meta package. The meta-analysis of dichotomous variables was performed using RevMan (version 5.4.1).

## Results

### Study characteristics

Four studies were included in this study (4, 19, 22, 23). This included one retrospective cohort study (*n* = 8,706) (4), one retrospective case-control study (*n* = 339) (22), one case series (*n* = 9) (19) and one case report (*n* = 1) (23). 164 out of 9,055 patients underwent pENPG across the studies. Two studies investigated pENPG as a single treatment arm (19, 23), whereas the other two studies compared pENPG to either IMRT (22) or open surgery (4). Two studies pooled patients who had undergone pENPG only with those who had received additional radiotherapy and or chemotherapy (4, 19). The study characteristics are summarised in Table 1. Two studies reported details on patient demographics (Table 1) (4, 22). Overall, 62 females and 92 males were treated with pENPG, with a combined mean (± SD) age of 56.3 ± 15.7 years.

**Table 1:** Study characteristics & patient demographics.

Study	Year	Country	Study design	Treatment groups (*n* = number of patients)	Age (mean, SD)	F:M
					Endoscopic only	
Finegersh et al. (4)	2022	United States	Retrospective cohort study	Endoscopic ± (C)RT (*n* = 144) Open ± (C)RT (*n* = 154) RT only (*n* = 8,408)	56.9 (15.6)	59:85
Liu et al. (23)	2020	China	Case report	Endoscopic only (*n* = 1)	n/a	n/a
Liu et al. (22)	2019	China	Retrospective case-control study	Endoscopic only (*n* = 10) RT only (*n* = 329)	47.0 (14.5)	3:7
Castelnuovo et al. (19)	2013	Italy	Case series	Endoscopic ± (C)RT (*n* = 9)	n/a	n/a

(C)RT, chemoradiotherapy; RT, radiotherapy; SD, standard deviation; F:M, number of females to males.

### NPC characteristics

Details on NPC characteristics can be found in Table 2. No studies included details regarding tumour size or dimensions. Two studies included information on tumour location (4, 23), with the posterior wall being the most common subsite (12%). All studies reported tumour stage using the Union for International Cancer Control/American Joint Committee on Cancer (UICC/AJCC) staging 7th Edition, either with tumour (T) and nodal (N) classifications (4), or the stage (either I, II, III or IV) (19, 22, 23). Out of the pENPG-treated patients, 58% had localised stage I pNPC (T1N0M0). Two studies grouped tumours histologically using the WHO classification (4, 22). As expected, undifferentiated NPC was the predominant histological type in the Asian cohort, but no studies included information about EBV status (22).

**Table 2:** Tumour characteristics for the endoscopic groups.

Study	Symptoms	Size (mm)	Anatomical subsite (n)	Staging	Histology
Finegersh et al. (4)	n/a	n/a	Superior wall (5) Posterior wall (36) Lateral wall (8) Anterior wall (8) Overlapping (10) Not specified (77)	T1 (84), N0 (81) T2 (20), N1 (30) T3 (21), N2 (27) T4 (22), N3 (6)	Carcinoma NOS (10) Carcinoma, undifferentiated (7) SCC (90)
Liu et al. (23)	n/a	n/a	Posterior wall (1)	Localised stage I NPC (1)	n/a
Liu et al. (22)	n/a	n/a	n/a	Localised stage I NPC (10)	WHO II (1) WHO III (9)
Castelnuovo et al. (19)	n/a	n/a	n/a	Stage I (5) Stage II (1) Stage III (2) Stage IV (1)	n/a

n, number of patients (in brackets); NOS, not otherwise specified; SCC, squamous cell carcinoma; WHO I, keratinised well-differentiated squamous cell carcinoma; WHO II, non-keratinising differentiated squamous cell carcinoma; WHO III, undifferentiated carcinoma.

### Operation details

Surgical selection criteria for pENPG were only described in one study (22). Criteria included (1) Tumour diameter less than or equal to 1.5 cm; (2) Distance of tumour margin to the internal carotid artery (ICA) of greater than or equal to 0.5 cm, and (3) Minimum axial diameter of less than or equal to 0.4 cm and 0.6 cm for retropharyngeal and cervical lymph nodes respectively. Intra-operative details were described in one study (22). Average operation length was 92.5 min (range 60–135) and average bleeding volume was 20 mL (range 10–100). Alongside pENPG, patients had reconstruction of the defect with a nasoseptal flap in two studies (*n* = 22) (19, 22) and neck dissection (*n* = 32) in one study (4). All operative details are summarised in Table 3.

**Table 3:** Operation details for primary endoscopic nasopharyngectomy.

Study	Selection criteria for surgery	Pre-op duration	OT length in minutes (range)	OT bleeding volume in ml (range)	Additional details (number of patients)
Finegersh et al. (4)	n/a	n/a	n/a	n/a	Neck dissection (32)
Liu et al. (23)	n/a	n/a	n/a	n/a	n/a
Liu et al. (22)	Tumour diameter ≤1.5 cm Distance from tumour to ICA ≥0.5 cm Axial diameter of lymph nodes ≤0.4 cm (retropharyngeal) & ≤0.6 cm (cervical)	n/a	92.5 (60–135)	20 (10–100)	Nasoseptal flap (6)
Castelnuovo et al. (19)	n/a	n/a	n/a	n/a	Nasoseptal flap (16)

Pre-op duration, Duration from diagnosis of primary NPC to surgical management; OT, operation; ICA, internal carotid artery.

### Surgical approach

**Figure 2:**
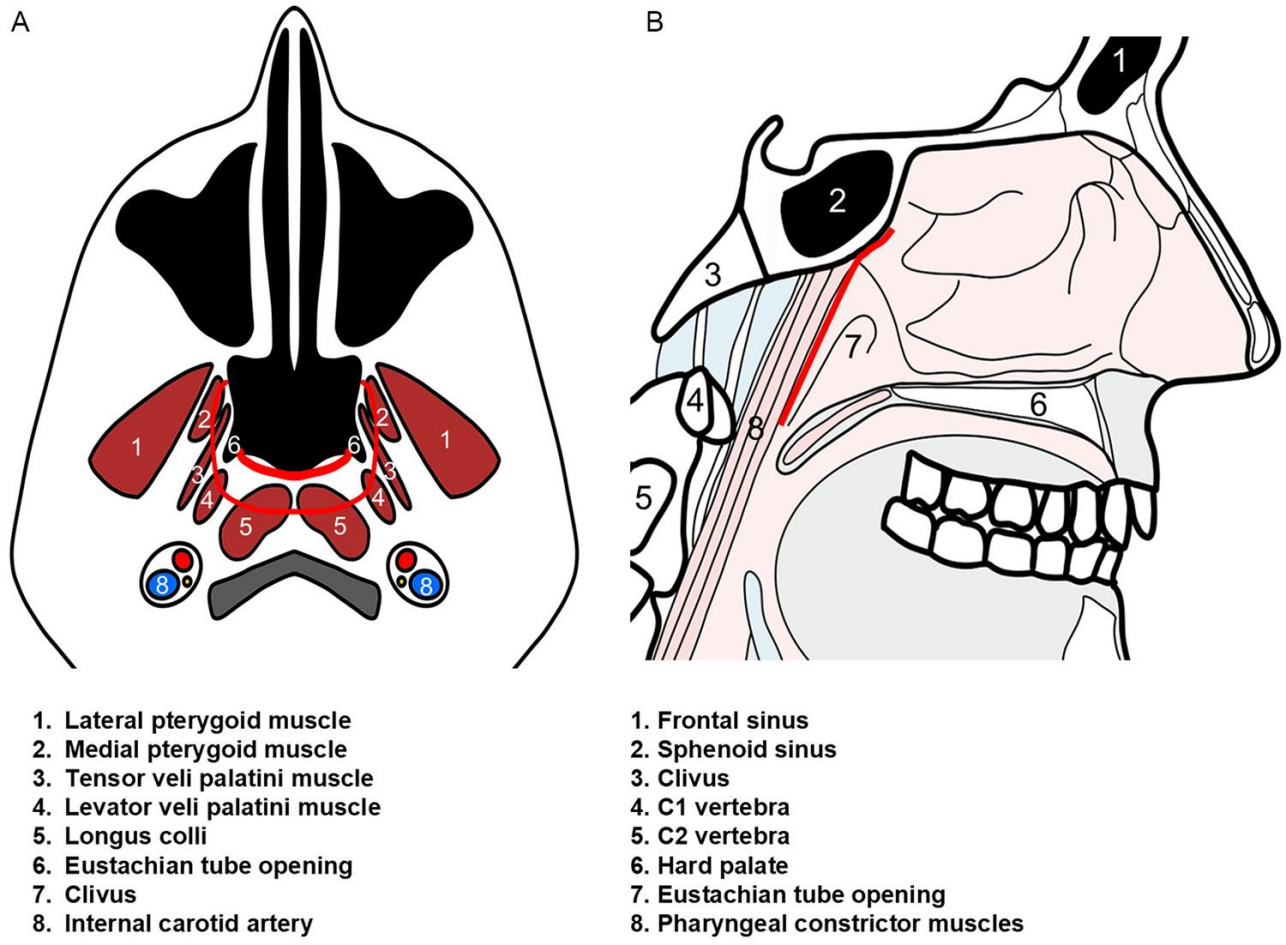
Castelnuovo classification of nasal endoscopic resection. **(A)** Axial view, depicting Type I and III NER (inner and outer red lines respectively). **(B)** Sagittal view, depicting Type II NER (red line).

Three studies describe their surgical method for pENPG (4, 22, 23). Two studies describe a method for en-bloc removal of the tumour (22, 23). Considering the NP as a cube, the anterior, superior, right lateral, left lateral, posterior and inferior walls are excised in that order. This results in a tumour surrounded by normal NP mucosa which can be removed in one go - a method they refer to as the “dumpling making technique”. Surgical margins were defined as the gross tumour volume (GTV) plus a 5–10 mm peripheral mucosal margin and a 2–3 mm skull base margin (sphenoid and clivus). Castelnuovo et al. describes three levels of nasal endoscopic resection (NER) according to the extent of resection required (Figure 2) (19). Type 1 NER is performed where disease is limited to posterosuperior NP wall. Type 2 NER is required for superior extension of the tumour to the anterior wall and floor of the sphenoid sinus. Type 3 NER is for lateral extension to the lateral NP, up to parapharyngeal space. The latter is the most invasive, involving an ipsilateral endoscopic medial maxillectomy and removal of the posterolateral NP using a transethmoid-pterygoid approach. Surgical margins were examined with frozen sections and resection was continued until clear margins or further resection was impossible due to invasion of critical structures.

### Survival outcomes following pENPG

**Figure 3:**
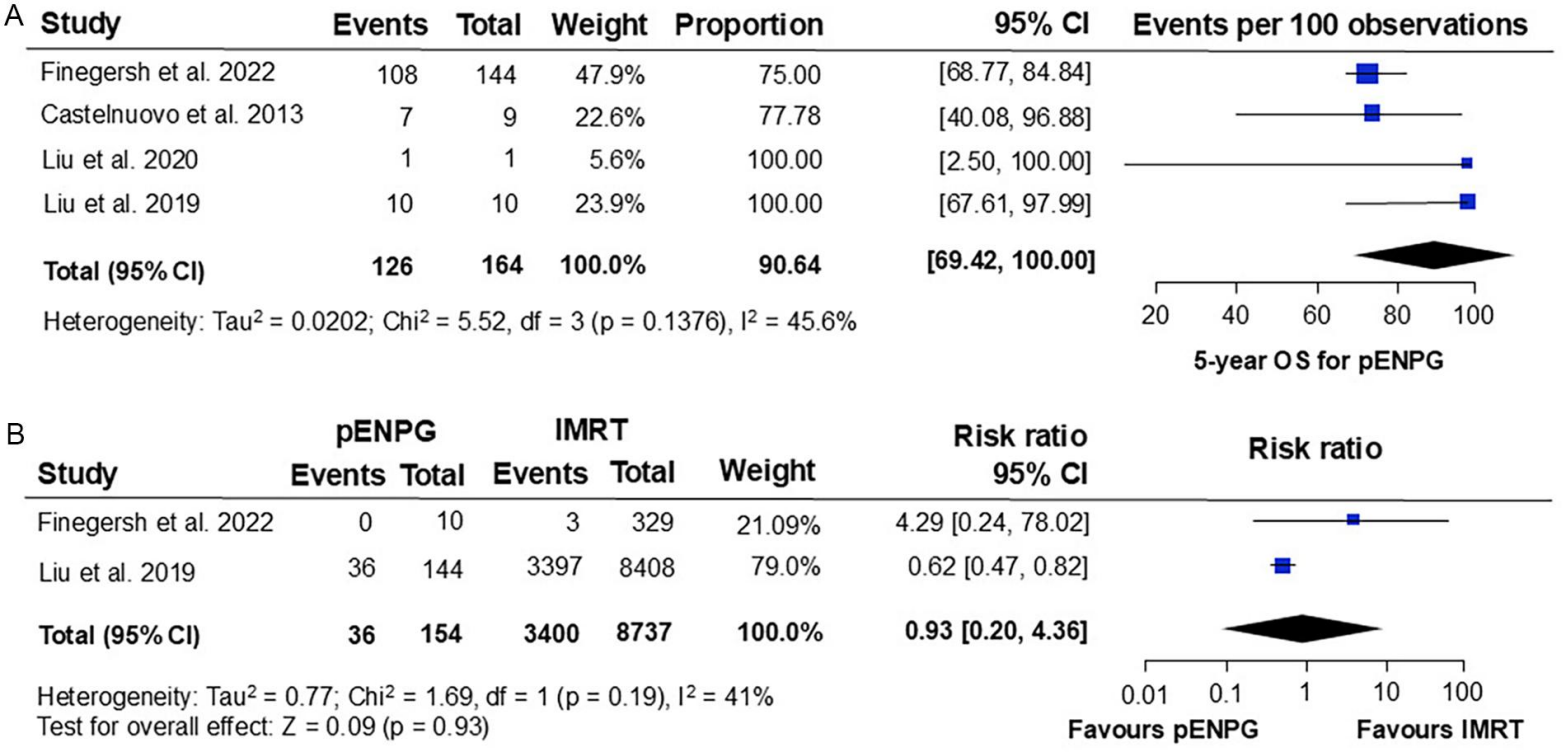
**(A)** forest plot of 5-year OS outcomes following pENPG for pNPC. **(B)** Forest plot of 5-year OS outcomes following pENPG compared with IMRT. CI, confidence interval; OS, overall survival.

Follow-up times range from three to five years and outcomes are summarised in Table 4. The primary outcome was five-year OS. For treatment of localised stage I pNPC (T1N0M0) using pENPG only, one study reported a 5-year OS of 100% (*n* = 10) (22) and another study reported a three-year OS of 100% (*n* = 1) (23). The two other studies treated varying stages of pNPC (stage I-IV) with pENPG alone or alongside radiotherapy and/or chemotherapy (4, 19) and 5-year OS was at 74.7% and 78% respectively. Secondary outcomes include other measures of survival, such as DFS, LRFS, RRFS and DMFS. Three-year DFS for pENPG-treated stage I pNPC was 100% (*n* = 1) (23), whereas 5-year DFS for pENPG-treated stage I-IV pNPC was 56% (*n* = 9) (19). 5-year LRFS, RRFS and DMFS were 100% for pENPG-treated stage I pNPC (*n* = 10) (22). We conducted a five-year OS meta-analysis following pENPG using a random effects model. The forest plot for this meta-analysis is illustrated in Figure 3A. The I^2^ statistic was 45.6%, suggesting a moderate level of heterogeneity between studies. Cochran's *Q* test yielded a *p*-value of 0.1376, exceeding the conventional threshold for detecting statistically significant heterogeneity (*p* < 0.05), but the test's ability to detect true differences may be limited by the small sample size. The pooled 5-year OS estimate was 90.6% (95% CI = 67.6–97.9). The pooled estimate and confidence intervals represented by the diamond does not cross the line of no survival, suggesting that pENPG has a statistically significant effect on survival.

**Table 4:** Outcomes data for primary endoscopic nasopharyngectomy.

Study	Follow-up time	OS (n)	DFS (n)	Other survival outcomes	Post-operative complications (n)
Finegersh et al. (4)	5 years	74.7% (108)	n/a	n/a	n/a
Liu et al. (23)	3 years	100% (1)	100% (1)	n/a	n/a
Liu et al. (22)	5 years	100% (10)	n/a	LRFS (100%) RRFS (100%) DMFS (100%)	Decreased dysphagia & xerostomia compared to RT
Castelnuovo et al. (19)	5 years	78% (7)	56% (5)	n/a	Occipital headache (8) Numbness of hard palate ipsilateral to resection area (2) Persistent glue ear with conductive hearing loss (4) Temporary masticatory impairment (3)

OS, overall survival; DFS, disease-free survival; n, number of patients; LRFS, local relapse-free survival; RRFS, regional relapse-free survival; DMFS, distant metastasis-free survival; RT, radiotherapy.

### Complications following pENPG

Post-operative complications were discussed by all studies. One study assessed quality of life outcomes between pENPG and RT-treated patients, finding that pain, dysphagia and xerostomia scores (using QLQ-C30 and QLQ-H&N35) were significantly reduced in the pENPG group compared to the RT group (22). Surgical complications of pENPG appeared to be associated with the extent of surgical resection (19). In Castelnuovo et al. (19), Type I NER and Type II NER were only associated with occipital headache (*n* = 3). Type III NER was associated with more complications, including occipital headache (*n* = 5), numbness of the hard palate ipsilateral to surgical site (*n* = 2), persistent glue ear with associated conductive hearing loss (*n* = 4) and temporary masticatory and swallowing impairment (*n* = 3) which did not persist in the long term.

### pENPG vs. IMRT outcomes

Liu et al. was the only study to directly compare survival outcomes among pENPG-treated and RT-treated patients with localised stage I pNPC (22). Five-year OS, LRFS, RRFS and DMFS was 100% with pENPG and 99.1%, 97.7%, 99.0% and 97.4% respectively with RT treatment. Kaplan–Meier survival analysis revealed no statistically significant difference between the two forms of treatment (*p* > 0.05). Finegersh et al. compared pENPG to IMRT as well, finding that the 5-year OS of the pENPG group was 74.7% compared with 59.6% in the RT group (4). However, 63.2% of the pENPG group were treated with adjuvant RT as well so direct inferences could not be made. A meta-analysis was performed using risk ratio (RR) as the effect measure and five-year mortality as the event outcome (as shown in Figure 3B). Between-study variability was moderate, with an I^2^ statistic of 41%, and heterogeneity was not statistically significant (Cochran's Q, *p* = 0.19); however, the small sample size limited the reliability of this assessment. The pooled analysis produced an estimated risk ratio (RR) of 0.93, indicating a favorable effect of pENPG over IMRT, although this result was not statistically significant as indicated by the diamond overlapping the line of no effect.

### Risk of bias

**Figure 4:**
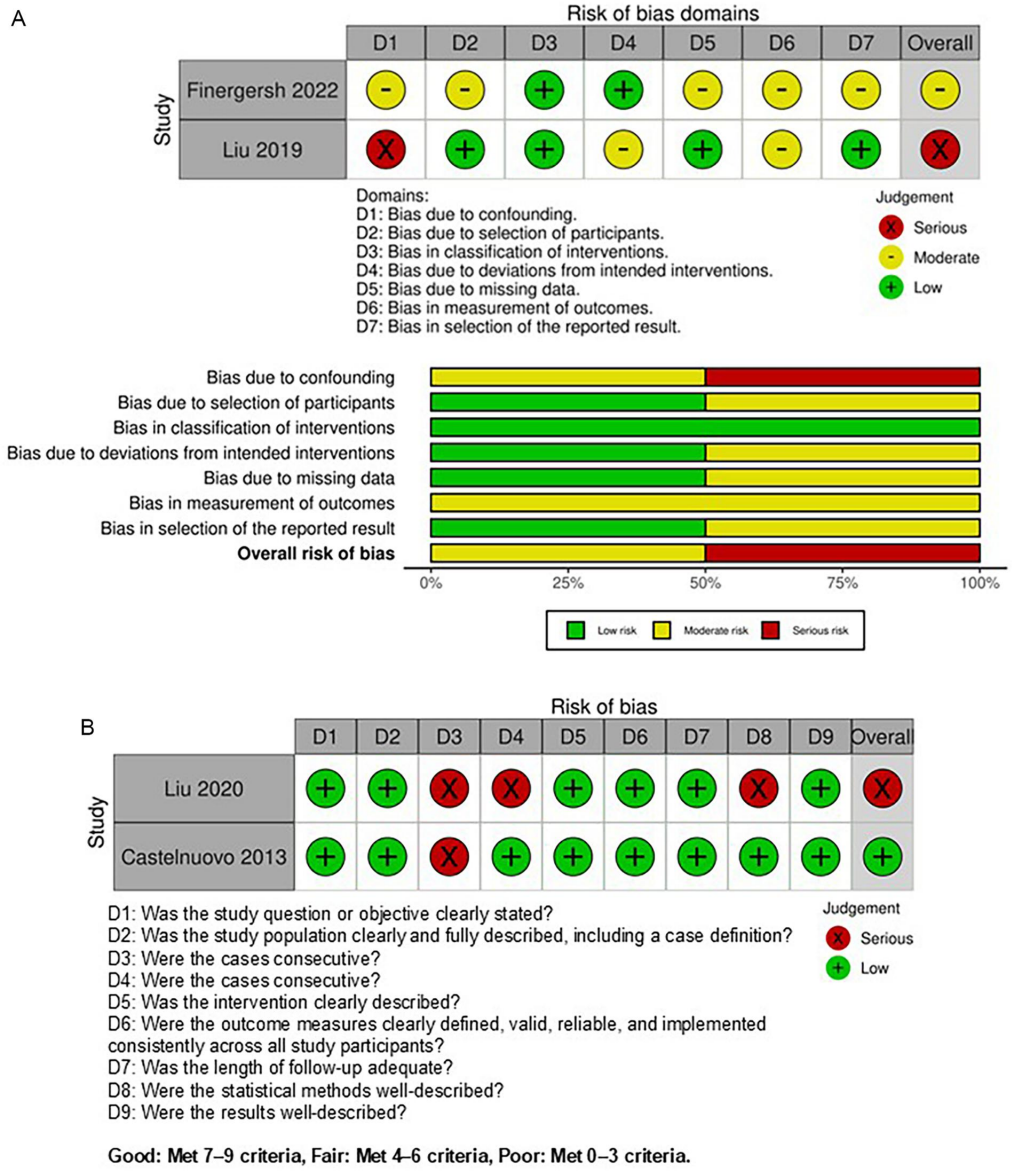
**(A)** results from risk of bias in nonrandomized studies of interventions (ROBINS-I) tool. **(B)** Results from NIH Quality Assessment Tool for Case Series Studies.

The ROBINS-I risk of bias assessment was used to assess one retrospective cohort study (4) and one retrospective case-control study (22). The NIH Quality Assessment Tool for Case Series was used to assess one case series (19) and one case report (23). The risk of bias in the interventional studies ranged from moderate to serious and the quality of case studies ranged from fair to good. Results are displayed diagrammatically in Figure 4.

## Discussion

This study provides a comprehensive evaluation of the existing literature on the use of pENPG for the treatment of pNPC. The number of existing studies utilising pENPG is sparse and only four studies were included in this analysis. Results from the meta-analysis revealed a pooled 5-year OS of 90.6% with pENPG for all stages, and 100% for the two studies that reported on stage I disease only. Unsurprisingly, pENPG was associated with significantly reduced RT-associated toxicities and the severity of surgical complications depended on the extent of resection. There is no clear consensus regarding the selection criteria for pENPG, but factors such as tumour size, proximity to important neurovascular structures and lymph node involvement are likely to play a significant role.

Only four studies were identified to have used solely pENPG for the treatment of pNPC. This demonstrates the current rarity of its use. Current ESMO-EURACAN guidelines recommend treatment of Stage I-II disease with RT alone and Stage III and IVa with CRT, with pENPG not being part of the standard guidelines (24). Although two studies recorded the histological type of their participants, neither study investigated the significance of this on clinical outcome, and no studies included data on EBV status. The vast majority of NPC cases in the endemic regions of Asia are EBV-associated undifferentiated NPC (2). There is building evidence that EBV is involved in all stages of undifferentiated NPC development, creating a tumour microenvironment that facilitates tumour growth and drug resistance (25). Plasma EBV-DNA levels have been shown to be a marker of tumour burden and is a strong predictor for relapse, and is also a target for novel immunotherapies. Pre-treatment EBV levels could be used to help individualise and de-escalate treatment and pENPG could play a potential role in this (26). Tumour subsites were reported by two studies, with the posterior wall being the most common site (4, 22). Knowing the tumour origin site is essential for informing feasibility of endoscopic resection. Excision of tumours that extend laterally to the parapharyngeal space or superiorly to the anterior cranial fossa are unlikely to be successful (27). Involvement of multiple subsites is correlated with more advanced disease and lymph node involvement (28), in which pENPG would also be unsuitable. Most pENPG patients had early disease, minimising the risks of surgery and providing maximal benefit (27).

Strict surgical selection criteria are essential to minimise risk, optimise outcomes and prevent conversion to an open approach (29). One criteria was a minimal axial diameter of < 0.4 cm for retropharyngeal nodes and < 0.6 cm for cervical nodes, the rationale being that more sizeable lymph nodes are highly sensitive for metastasis (23). Other criteria included having a minimum safe margin (e.g., at least 0.5 cm away from the ICA) and operating on smaller tumours (< 1.5 cm) only. Absolute contraindications would include encasement of the ICA, intradural involvement, intracranial extension or distant metastasis (19). Parapharyngeal extension may not exclude pENPG, as some centres have performed sENPG on patients with significant parapharyngeal involvement (30). Patient factors that would exclude surgery include significant co-morbidities, being medically unfit or intolerance to general anaesthesia (29). Intraoperative details were only reported by one study, with minimal complications noted during the operation and average bleeding volume of 20 mL (22). The most commonly used reconstructive flap for pENPG was the pedicled nasoseptal flap vascularised by the sphenopalatine artery (Hadad-Bassagasteguy flap) (19, 22), which has become the workhorse for reconstruction following endoscopic nasal procedures. Its advantages include its thinness and pliability, the ease of harvesting and the lack of requirement of an external incision (31). However, its use may be complicated by tumour involvement of the nasal septum and its thinness offers less protection to critical structures if further radiation is required. Alternative flaps that could be considered include the submental island flap, temporalis muscle flap or free flaps such as the radial forearm flap (31).

The primary outcome was three and five-year OS post pENPG. For the treatment of early-stage pNPC (T1N0M0) with pENPG, one study reported a 5-year OS of 100% (*n* = 10) (22) and another study reported a 3-year OS of 100% (*n* = 1) (23). For secondary outcomes, 5-year LRFS, RRFS, DMFS and 3-year DFS were also 100% over the two studies (22, 23). These findings are similar to survival outcomes in patients treated with IMRT alone. A retrospective analysis of 198 early-stage pNPC (T1-T2bN0-1M0) patients who underwent IMRT alone revealed a 5-year estimated DSS, LRFS and DMFS of 97.3%, 97.7% and 97.8% respectively (8). For T1N0 lesions specifically, 5-year LRFS and DMFS were both 100%. Long term follow-up of pENPG patients beyond five years would be useful to assess whether survival outcomes are sustained. A retrospective study with 15-year follow-up of early-stage (T1-2N0-1M0) pNPC patients treated with IMRT ± chemotherapy showed 10-year LRFS, DMFS, DSS and OS of 93.3%, 93.5%, 92.9% and 88.2% respectively (32), indicating that long-term protection is provided by chemoradiotherapy. Survival outcomes with pENPG for locoregionally advanced pNPC (T2b-4) is less clear. Two studies in our review investigate the treatment of stage I-IV pNPC with pENPG alone or in combination with chemo/radiotherapy (4, 19). However, the results were pooled and did not differentiate between different tumour stages or the specific treatment received. 5-year OS for this pooled group of patients was 74.7% and 78% respectively, expectedly lower than early-stage pNPC cohorts. These results are comparable to 5-year OS outcomes of locoregionally advanced pNPC patients who had undergone CCRT alone (33). Our pooled results from meta-analysis of all disease stages revealed a 5-year OS of 90.6% with pENPG and a RR of 0.93 compared with IMRT, supporting the conclusion that pENPG could be a feasible alternative to IMRT.

IMRT is the current standard for treatment of early-stage pNPC due to its safety and efficacy profile. Toxicities are significantly reduced compared to previous techniques such as conventional two-dimensional radiotherapy (34). NTCP models developed for predicting xerostomia, dysphagia and tube-feeding dependence reveal that significant predictors include dose-volume of irradiation to salivary glands swallowing structures (e.g., pharyngeal constrictor muscles) as well as advanced T-stage and positive N-stage (11, 12, 35). For early stage disease, IMRT-related toxicities are generally mild. A retrospective analysis of 198 early-stage pNPC (T1-T2bN0-N1M0) patients treated with IMRT alone found that the most common acute toxicities were at Grade 1 or 2 (8). Surgical complications for early-stage NPC also appear to be mild, with one study reporting occipital headache as the only complication of type I NER (22). In one of the included studies on 10 patients with stage I NPC, subjective self-reported scoring of pain, swallowing, dry mouth and sticky saliva in QLQ-C30 and QLQ-H&N35 questionnaires was shown to be slightly better for pENPG compared with IMRT although more quantitative evidence is needed to support the above (22).

Radiation toxicities are increased for late stage disease with the gold-standard treatment being high dose radiotherapy and concurrent chemotherapy. One study showed that for patients with stage III-IVb disease, 63.2% developed one or major late toxicities, including temporal lobe necrosis in 54% and xerostomia in 87.7% (13). Radiotherapy-induced vascular injuries include carotid blowout syndrome (36) and vertebral artery stenosis (37), both of which carry significant morbidity and mortality. Osteoradionecrosis of the skull base and cervical vertebrae are life-threatening complications that can be complicated by CNS infection and atlantoaxial instability and are very challenging to treat (38, 39). It is important to note that these are rare complications occurring in advanced cases, associated with significant radiation doses (± re-irradiation) and are often inoperable upon initial presentation.

Recent advancements in radiation oncology extending beyond IMRT may further enhance outcomes of radiotherapy for NPC. Modern precision radiotherapy techniques include volumetric-modulated arc therapy, intensity-modulated proton radiotherapy (IMPT) and intensity-modulated carbon-ion radiotherapy (IMCT) (40). The latter two techniques are forms of particle radiotherapy (compared to the conventional photon radiotherapy) that exploits “Bragg peak” characteristics to provide a highly targeted dose and minimises exposure to surrounding healthy tissue. One case-control study reports lower rates of grade 2 + acute toxicities with IMPT compared with IMRT (40.3% vs. 52.7% respectively over 55.4 month median follow-up) and no statistically significant differences in 5-year recurrence rates and overall survival (41).

The management of regional neck lymph nodes after pENPG for pNPC remains unclear. This topic was not discussed in significant detail by any of the included studies. In Finegersh et al., a greater proportion of open surgery patients underwent either additional radiotherapy and/or neck dissection compared with pENPG patients, but indications or outcomes following either treatment were not described (4). For Liu et al., the IMRT group underwent bilateral irradiation of upper neck lymph node groups, whereas the pENPG group was managed with surveillance only (taking into account strict selection criteria was imposed including minimum axial diameter of retropharyngeal lymph nodes < 0.4 cm and cervical lymph nodes < 0.6 cm), with a LRFS, RRFS and DMFS of 100% in both groups (22). In Castelnuovo et al., an unclear proportion of pENPG patients underwent neck irradiation, but none had neck dissection (19). Liu et al. does not mention the management of neck nodes (23). Current international standards, including ESMO-EURACAN, recommend bilateral neck irradiation of nodal levels II-V and the retropharyngeal lymph nodes (level VII) at the minimum for node-negative disease (24). This is based on research findings that cervical lymph node metastases follow an orderly manner, starting from most commonly the retropharyngeal lymph nodes, followed by levels II, III and Va, and then finally to the lower neck (IV and Vb) (42). More recently, a randomised phase III trial found non-inferiority of selective upper neck irradiation (irradiation of levels II, III and Va in the uninvolved neck, excluding levels IV and Vb) in three-year RRFS (97.7% vs. 96.3%) as well as reduced late toxicities compared with whole neck irradiation, including any-grade hypothyroidism (30% vs. 39%), skin toxicity (14% vs. 25%), dysphagia (17% vs. 32%) and neck tissue damage (23% vs. 40%) (43). These findings were incorporated into a recent international consensus and contouring atlas which suggests that uninvolved lower neck levels IV & Vb may be omitted in select cases (44). Neck dissection could also be a feasible alternative to RT for elective nodal management following pENPG, and the treatment modality should be determined on a case-by-case basis depending on the individual and based on discussions by an experienced multidisciplinary team within a high-volume tertiary centre. RT could also be retained as a salvage option following close surveillance or neck dissection after pENPG. Future studies should investigate these potential options to develop optimised strategies.

There remains significant clinical complexity regarding the use of endoscopic surgery compared with radiotherapy for pNPC. pENPG avoids radiation toxicities and preliminary research suggests minimal surgical complications. However, in early stage disease, radiotherapy toxicities are generally mild (45). Advancements in radiotherapy techniques, including proton therapy, may further mitigate against radiation toxicities (46). Current evidence remains insufficient to support pENPG over radiotherapy for resectable disease. Therefore, further research, particularly prospective studies, are required to better define the role and utility of endoscopic surgery for pNPC.

Our study is limited by a small number of included studies. The number of pENPG patients are far outweighed by those undergoing IMRT and selection bias is likely, limiting the generalisability of the findings. The existing studies often rely on retrospective data, often with small sample sizes and inconsistent reporting standards. The inclusion of EBV-DNA data for undifferentiated cases and exploration into the management of regional neck lymph nodes following pENPG in future studies would be beneficial. Currently, no randomised control trials exist which compare pENPG to IMRT, however, one prospective observational study of this kind is currently ongoing (NCT03353467) (47).

## Conclusion

The current utilisation of pENPG for pNPC is rare among standard practice. pENPG for early-stage pNPC may be a feasible alternative to IMRT alone, providing reductions in IMRT-related toxicities, but evidence for its use is currently sparse. If endoscopic approaches to NPC are to become more widely adopted, prospective studies with larger sample sizes and extended follow-up periods would be beneficial.

## Data Availability

The original contributions presented in the study are included in the article/Supplementary Material, further inquiries can be directed to the corresponding author.
